# Supplementary material for: PML/RARa Interferes with NRF2 Transcriptional Activity Increasing the Sensitivity to Ascorbate of Acute Promyelocytic Leukemia Cells
Source: Cancers (Basel). 2019 Dec 30;12(1):95. doi: 10.3390/cancers12010095 (PMC7016898; doi:10.3390/cancers12010095)
Supplement: Supplementary file 1 [file cancers-12-00095-s001.zip › supplementary files/supplementary tables.docx]

**Supplementary Materilas**

**Table S1.** Anti-human antibodies used.

| **Antigen** | **Purchased from** | **Used** |
| --- | --- | --- |
| anti NRF2 ab62352 | Ab Cam, (Cambridge, UK) | WB-IP-CHIP-IF |
| A-Tubulin | Abcam, (Cambridge, UK) | WB |
| B-Actin | Cell Signaling Technology, (Beverley, MA, USA) | WB |
| anti Keap1 | Flarebio biotech, (Baltimore, USA) | WB |
| RARa (C20, sc551) | Santa Cruz Biotechnology, Inc. (Dallas, USA) | IP |
| Histone H3 ab1791 | Abcam, (Cambridge, UK) | WB |
| Normal rabbit IgG | Cell Signaling Inc. (Massachusetts, USA) | CHIP |
| PML (H-238, sc5621) | Santa Cruz Biotechnology, Inc. (Dallas, USA) | WB-CHIP |

WB: western blot; ChIP: Chromatin Immunoprecipitation; IP: Immunoprecipitation.

**Table S2.** Primer List.

| **Q-PCR Primers** | |
| --- | --- |
| **ABL:** | Fow: CGCTATTCGCAATCAGGGTTA |
|  | Rev: GGGCGTGTGACCGTAGCT |
| **HMOX1** | Fow: CCAGCAACAAAGTGCAAGATTC |
|  | Rev: CCACCAGAAAGCTGAGTGTAAG |
| **NQO-1** | Fow: CCGTGGATCCCTTGCAGAGA |
|  | Rev: AGGACCCTTCCGGGAGTAAGA |
| **AKR1C1** | Fow: GGCCTAAACAGAAATGTGCG |
|  | Rev: CATCCTCTGTGTCACCATCC |
| **CHIP Primers** | |
| **HMOX1** | For: CCATCTGGCGCCGCTCTGC |
|  | Rev: GAGCAGCTGGAACTCTGAGGA |
| **GAPDH** | Fow: GTATTCCCCCAGGTTTACAT |
|  | Rev: TTCTGTCTTCCACTCACTC |
